# Calorimetric characterization of the stability and activity of trimethylamine‐N‐oxide (TMAO) demethylase from *Methylocella silvestris* BL2

**DOI:** 10.1002/pro.70364

**Published:** 2025-10-28

**Authors:** Federico Cappa, Nakia Polidori, Daniele Giuriato, Danilo Correddu, Arianna Marucco, Sheila J. Sadeghi, Renzo Levi, Gianluca Catucci, Gianfranco Gilardi

**Affiliations:** ^1^ Department of Life Sciences and Systems Biology University of Torino Torino Italy

**Keywords:** AlphaFold, differential scanning calorimetry, isothermal titration calorimetry, TMAO, TMAO‐demethylase

## Abstract

Trimethylamine‐N‐oxide (TMAO) is an organic osmolyte found in numerous species and is known to have a range of biological effects. TMAO has recently garnered attention in the medical field due to its association with cardiovascular diseases, underscoring the need for its reliable detection and quantification. Current methods for TMAO analysis often rely on hazardous reagents or costly analytical instrumentation. In this study, we focus on *Methylocella silvestris* BL2, which produces a TMAO‐demethylase (Tdm), with the aim of developing a direct enzymatic assay for TMAO detection. We report on the bioinformatic analysis, expression, purification, and calorimetric characterization of Tdm. Structural predictions generated by AlphaFold suggest that the protein, previously described as hexameric, is organized as a trimer of dimers. The 3D model reveals that the binding sites for the metal cofactors Zn^2+^ and Fe^2+^ are located in close proximity. Differential scanning calorimetry (DSC) experiments show an irreversible unfolding behavior with two independent endothermic transitions, consistent with a two‐state model. Isothermal titration calorimetry (ITC) was employed in a time‐resolved manner to determine the enzyme's optimal reaction pH and substrate detection limit. The assay revealed an optimal pH of 7.0, a minimum effective enzyme concentration of 100 nM, and a TMAO detection limit of 10 μM. Kinetic parameters were also precisely measured using ITC, with the highest observed *k*_cat_ value being 15.47 s^−1^ at 100 nM Tdm concentration. Overall, these findings support the potential application of Tdm as a sensitive and direct tool for the detection and quantification of the medically relevant biomarker TMAO.

## 1 INTRODUCTION

Nitrogen‐containing organic compounds play a fundamental role in living organisms (Somero, 2003). Among these compounds, amines like trimethylamine‐*N*‐oxide (TMAO) are very important both in animal (Shimizu & Smith, 2017; Yancey et al., 1982; Zerbst‐Boroffka et al., 2005) and prokaryotic kingdoms (Lidbury et al., 2014; Roberts, 2004; Yancey, 2004). In marine environments TMAO is a key osmolyte for animals like Chondrichthyes (Withers et al., 1994; Yancey & Somero, 1979; Yancey & Somero, 1980) and Osteichthyes (Gillett et al., 1997; Niizeki et al., 2002; Raymond, 1998; Raymond & DeVries, 1998; Samerotte et al., 2007; Yancey et al., 2014; Yancey & Somero, 1979), where it either counteracts high plasmatic levels of urea (Treberg et al., 2006) or it works as a piezolyte (Manisegaran et al., 2019), offsetting the hydrostatic pressure stress on membranes and proteins (Jethva & Udgaonkar, 2018). All these functions arise from the interactions established between TMAO and water molecules, rather than its direct interaction with macromolecules (Larini & Shea, 2013; Sasaki et al., 2016; van der Vegt & Nayar, 2017; Zetterholm et al., 2018). In humans, this amine has recently gained medical relevance due to the correlation between its high plasmatic concentration and the increased probability of cardiovascular diseases (CVD) (Tang et al., 2013). It is still to be understood whether TMAO is responsible for the mechanism causing CVDs or whether it is a consequent biomarker (Janeiro et al., 2018), but the importance of detecting and quantifying its presence in the progression of CVD is indubitably important. Commonly used methods to detect its presence are chromatography techniques, which are expensive and require specialized skills. Other methods involve the use of hazardous compounds and complex long procedures. Therefore, it is still of vital importance to develop a quick, user‐friendly and safe method to measure the TMAO.

**FIGURE 1:**
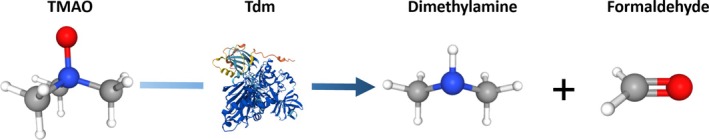
Reaction scheme of Tdm. The figure represents the reaction scheme of Tdm, in which TMAO is directly metabolized by the enzyme into formaldehyde and dimethylamine in a ratio of 1:1.

In this respect, *Methylocella silvestris* BL2, a soil bacterium (Dunfield et al., 2003), was recently reported to include a TMAO demethylase (Tdm) in its genome (Zhu et al., 2014). This bacterial enzyme is capable of metabolizing trimethylamine‐N‐oxide to dimethylamine and formaldehyde in a reaction with a 1:1 stoichiometry (Figure 1). Surprisingly, this enzyme can tackle this reaction both in the presence or absence of oxygen (Zhu et al., 2016). Its activity in anaerobiosis is most likely due to the fact that it can utilize the oxygen atom present in TMAO, thus not depending on external oxygen sources. Despite its great potential in biotechnology the crystal structure of Tdm is not known and the enzyme has been only partially characterized (Zhu et al., 2016).

In this work, we expressed and purified Tdm and we used differential scanning calorimetry (DSC) to map enzyme function and overall stability. We used AlphaFold (Evans et al., 2022; Jumper et al., 2021) to generate 3‐dimensional models of the protein and performed metal atom docking through the MIB2 server (Lin et al., 2016; Lu et al., 2012, 2022), highlighting previously neglected binding characteristics. Finally, since the demethylation reaction of Tdm is normally studied by indirect quantification of formaldehyde produced, we designed an innovative methodology to directly measure its product formation. The methodology is based on isothermal titration calorimetry (ITC) and it can be exploited for the direct measurement of kinetic parameters by correlating heat flow variation to the reaction rate (Catucci et al., 2019). This assay can work in either single injection or multiple injection mode, obtaining data useful for basic biochemical characterization of the optimal reaction pH or the calculation of kinetic parameters associated with the demethylation reaction.

## 2 RESULTS

### 2.1 Structure and fold prediction for Tdm

**FIGURE 2:**
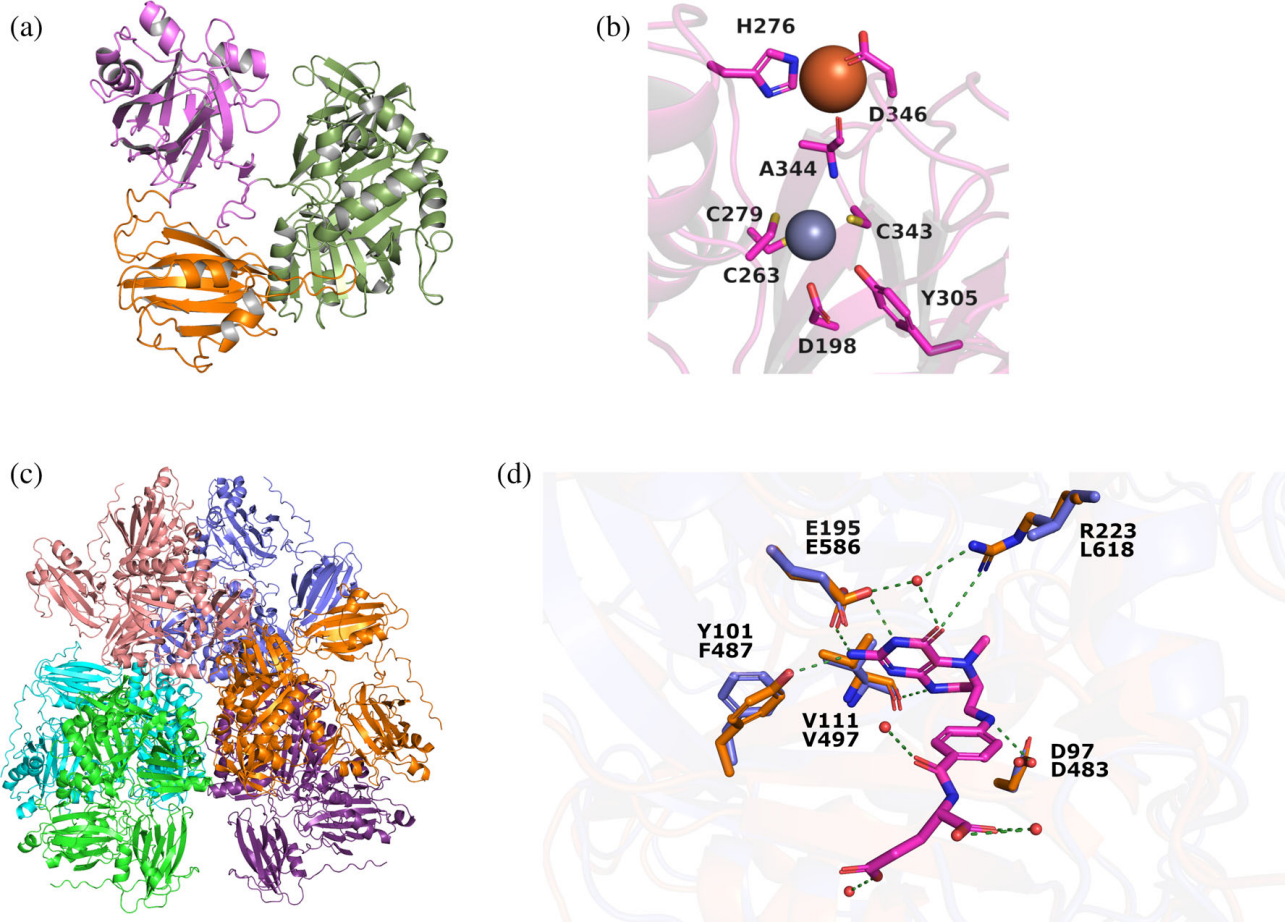
Tdm tri‐dimensional reconstruction obtained with AlphaFold and metal cofactors binding sites. (a) AlphaFold model of Tdm, divided in N‐terminal DUF1989‐like domain (orange), DUF1989 domain (magenta), and GCV_T domain (green). (b) Proposed metal binding sites for Zn^2+^ (gray) and Fe^2+^ (brown). (c) AlphaFold model of the putative quaternary structure of Tdm. (d) THF cofactor binding residues comparion between *Methylocella silvestris* Tdm and *Escherichia coli* K‐12 Gcv_T (PDB: 3A8I). Key substitutions of R223 and Y101 present in *E. coli* K‐12 with L618 and F487 in *M. silvestris* leading to the impossibility of THF cofactor coordination.

As no crystal structure of Tdm has been reported so far, AlphaFold (Evans et al., 2022; Jumper et al., 2021) was used to generate a 3D model. With the exception of some regions in the first 150 residues, the model displays high levels of confidence, according to pLDDT. As reported by Zhu et al. (2016), the enzyme has an N‐terminal domain bearing a DUF1989 fold and a C‐terminal domain homologous to the T protein of the glycine cleavage system (Gcv_T). The structure includes a third unreported domain at the N‐terminus (Figure 2a). The overall fold of this domain is similar to a DUF1989, but it lacks the zinc‐binding cysteines present in the other known structures (PDBs: 3SIY, 3ORU, 3DI4) and indeed it is not recognized as such by InterPro (Paysan‐Lafosse et al., 2023). Multiple sequence alignment (MSA) shows great variability in this N‐terminal sequence (Figure S1); however, this domain is ubiquitously present in Tdm enzyme homologs.

Tdm is known to bind Fe^2+^ and Zn^2+^. The binding site for the Zn^2+^ is formed by three cysteine residues (C263, C279, and C343) and it is easily identifiable within the DUF1989 domain because of its high structural similarity to other DUF1989 proteins. Zhu et al. (2016) proposed through mutagenesis experiments that H276 is coordinating the iron atom, while D198 acts as a bridging residue between Zn^2+^ and Fe^2+^. In the model obtained by AlphaFold, the zinc atom sits right in between these two residues, making the last assumption unlikely (Figure 2b). However, mutagenesis experiments in the zinc‐binding site also affected the binding of Fe^2+^, suggesting a significant relationship between the two binding sites. This can be interpreted as an indication that the two metal atoms bind in close proximity within the molecular structure. The MIB2 (Lin et al., 2016; Lu et al., 2012, 2022) server was used to explore possible iron binding sites and dock in them the Fe^2+^ atom. A possible binding site close to the zinc atom was identified and as proposed by Zhu et al. (2016), the iron is here coordinated by the side chain of H276, together with the one of D346 and the backbone of A344 (Figure 2b). MSA confirmed that H276 and D346 are highly conserved, while A344 is often replaced by threonine or proline (Figure S2). Tdm has been reported to be a hexamer in the native state, but no elution profile was provided at the time. Analytical size‐exclusion chromatography (Figures S2–S3, Table S1) revealed an elution profile consistent with a molecular weight in the range of 682 kDa (octamer) to 427 kDa (pentamer). Since the calibration curve is prepared using globular proteins, an exact evaluation of the oligomeric state of Tdm is not possible. However, the overall elution profile supports the presence of a complex oligomeric state, in line with the hexameric assembly proposed by Zhu et al. Several single amino acid substitutions on the DUF1989 domain were proven to alter the quaternary structure of the enzyme (Zhu et al., 2016). We built five AlphaFold (Evans et al., 2022; Jumper et al., 2021) models to explore the putative quaternary structure of Tdm. All the hexameric models form a trimer of dimers, with the dimers being almost identical in the five models (Figure 2c). A significant part of the dimerization interface is formed by the DUF1989 domain, which is in line with the alteration of the quaternary structure reported by Zhu et al. during their mutagenesis experiments (Zhu et al., 2016).

The C‐terminal domain of Tdm displays sequence homology with several proteins having a Gcv_T‐like fold and a structure deposited in the Protein Data Bank. Gcv_T from B*acillus subtilis* (PDB: 1YX2) has the highest homology (33.63%), followed by Gcv_T from *Thermotoga maritima* (30.56%), dimethylglycine oxidase of *Arthrobacter globiformis* (30.50%), and Gcv_T from *Escherichia coli* K‐12 (30.28%). The latter has been chosen for comparison, being the most characterized in literature. Gcv_T proteins use tetrahydrofolate (THF) as a co‐substrate to catalyze the demethylation of the glycine‐derived aminomethyl moiety attached to the H‐protein (Okamura‐Ikeda et al., 2010; Teplyakov et al., 2004). By comparing the Tdm model to the structure of Gcv_T from *Escherichia coli* K‐12 (PDB: 3A8I), it was noticed that Tdm lacks some key residues necessary for the interaction with this co‐substrate. R223 in *E. coli* Gcv_T forms a direct hydrogen bond with the THF and additionally coordinates a water molecule with E195, which is substituted by leucine in Tdm; additionally, the interaction of THF with Y101 is disrupted in Tdm by the substitution of this residue with phenylalanine (Figure 2d). Gcv_T is known to spontaneously produce formaldehyde in the presence of excessive substrate or in the absence of THF (Okamura‐Ikeda et al., 2010; Teplyakov et al., 2004); THF supplementation in Tdm slightly reduces formaldehyde release (Zhu et al., 2016), suggesting that THF binding is still present. THF is not necessary for TMAO demethylation; consequently, this co‐substrate usage is probably a vestigial remnant derived from the Gcv_T fold.

### 2.2 Expression and purification of Tdm

The Tdm gene was cloned into a pET28a(+) plasmid vector and expressed in *E. coli* BL21. Protein purification was carried out using a nickel affinity column exploiting the hexa‐histidine tag at the N‐terminus of the protein. Therefore, for protein elution we used increasing concentrations of imidazole. Elution from the nickel affinity column resulted in two different pools corresponding to either 150 mM (pool A) or 250 mM imidazole (pool B). The SDS‐PAGE gel of the eluted fractions shows a band around 85 kDa (Figure S4), in accordance with the predicted molecular weight of 83.8 kDa. After buffer exchange, the activity of the two eluted proteins was tested using formaldehyde quantification and only the pool eluted with lower imidazole was active. The loss of activity observed in the fraction eluted with 250 mM imidazole (Pool B) likely results from partial misfolding or destabilization of the protein structure caused by the higher imidazole concentration during elution, which can interfere with the maintenance of native tertiary/quaternary interactions required for activity.

### 2.3 Thermal stability and unfolding process of Tdm and its domains

**FIGURE 3:**
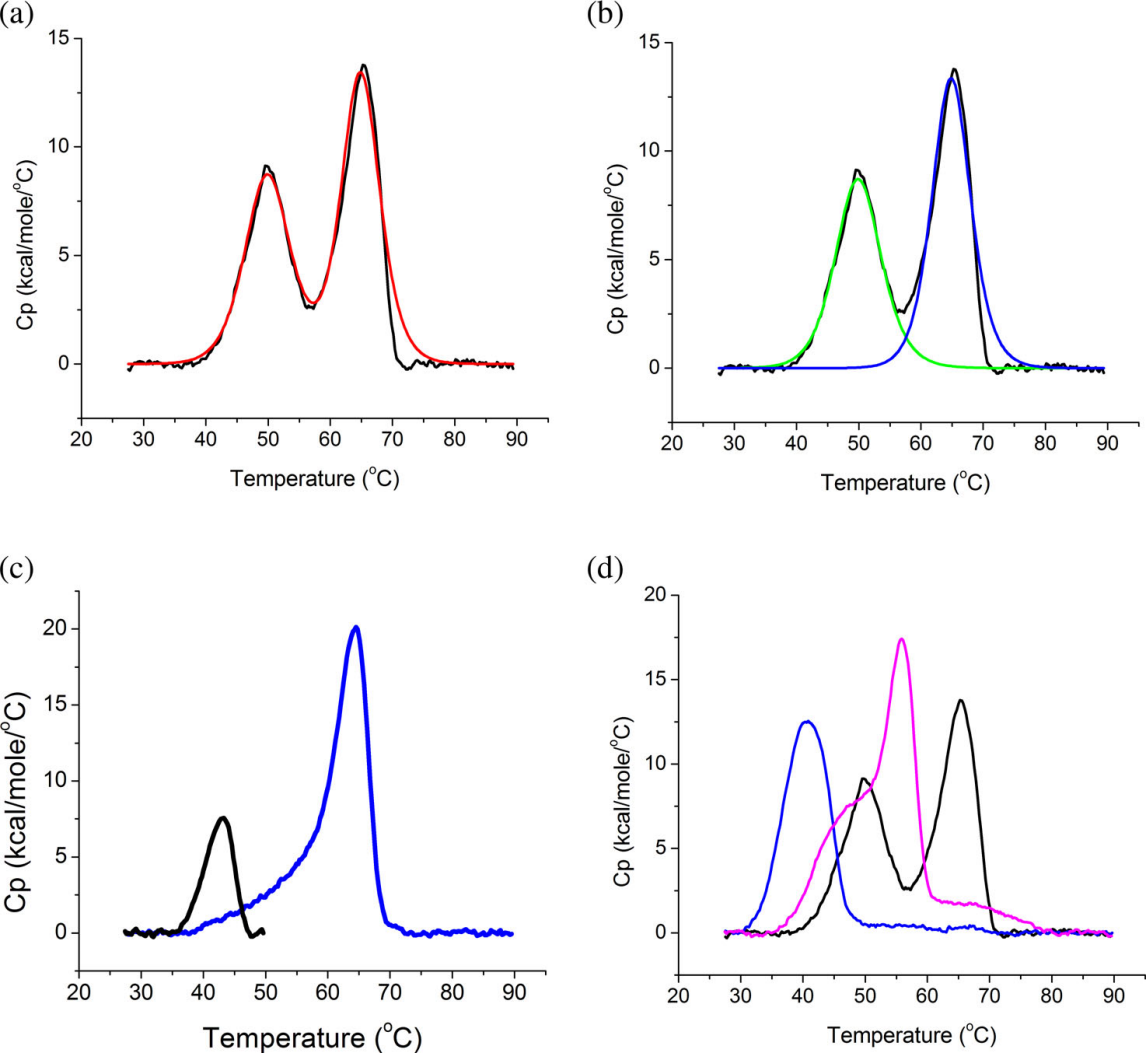
Differential Scanning Calorimetry of Tdm. (a) Thermogram with the experimental curve (black) and fitting of the two‐state denaturation model (red). (b) Two‐state deconvolution model of the first peak (green) and the second peak (blue curve) fitted on the experimental curve (black). (c) Denaturation in two steps (from 25 to 50°C (black), then from 25 to 90°C (blue)). (d) Thermogram of the two truncated versions compared to the full protein (Gcv_T‐like is the blue line; N‐terminal is the pink line).

DSC of purified Tdm was used to study its unfolding process. A temperature ramp increasing from 25 to 90°C was applied to a solution of pure Tdm, using a scan rate of 90°C/h. The thermogram resulting from the protein denaturation shows two endothermic peaks (Figure 3a). The fact that only two unfolding events are detected in the DSC experiments implies that two domains, probably the two N‐terminal ones, are denaturing cooperatively, acting as a homodimer within the 3‐domain protein. To elucidate this hypothesis, we expressed and purified both TdmΔ_1‐376_ (Gcv_T‐like) and TdmΔ_377‐761_ (N‐terminal) domains of the protein and measured their denaturation profile. Figure 3d displays the thermogram of the two truncated versions compared to the full protein. The Gcv_T‐like domain denatures at the lowest temperature, while the N‐terminal domain shows a shoulder around 48°C before a sharp peak at approximately 66°C. The thermogram of the two truncated versions was not altered when the two fractions were incubated together (not shown), indicating that the two domains alone are unable to interact. Furthermore, the melting temperature of the two domains is abundantly lower than the peaks observed for the full protein, suggesting that oligomerization plays a key role in the stabilization of the full protein. Cooling of the sample and rescan did not result in any endothermic transition indicating the irreversibility of the unfolding process. Since the molar enthalpy change per mole of cooperative unit (Δ*H*_VH_) is either equivalent to or greater than the total energy uptake (Δ_Cal_) for both peaks in the thermogram (Table 1), it can be stated that the enzyme denaturation process follows a two‐state mode, that is, directly transitioning from a native to an unfolded polypeptide without intermediate forms (Figure 3a,b) (Johnson, 2013). The thermogram contains two almost fully separated peaks; therefore, we hypothesized the presence of two independent unfolding events. For this reason, the protein solution was heated from 25 to 50°C, then the temperature was brought back to 25°C and heated up to 90°C again with a scan rate of 90°C/h. While the first domain cannot refold upon reheating the solution, the second peak is not affected by the unfolding of the first one, indicating an independent folding for the two endothermic transitions (Figure 3c). Furthermore, this setup was also used to denature the domains within Tdm for the ITC measurements.

**TABLE 1:** DSC values of endothermic peaks (*T*_m_), total energy uptake (Δ*H*_Cal_) and molar enthalpy change (Δ*H*_VH_).

Peak	Endothermic heat	Total energy uptake	Molar enthalpy change
	*T*_m_	Δ*H*_Cal_	Δ*H*_VH_
1	50.12 ± 0.07°C	86.1 ± 1.37 Kcal/mol	83.2 ± 1.66 Kcal/mol
2	65.00 ± 0.04°C	98.7 ± 1.17 Kcal/mol	127.0 ± 1.87 Kcal/mol

### 2.4 Activity assay of Tdm and its domains

To test if the purified enzyme was active, we incubated the enzyme for 60 min in the presence of 100 μM of TMAO and measured the released formaldehyde using a commercial kit (Sigma‐Aldrich). We additionally performed this assay on the isolated Gcv_T‐like and N‐terminal domains to test if any of them were active on their own. Figure S5 shows the calibration curve of the assay, while the results are shown in Table S2 As can be observed, Tdm generated an equimolar amount of formaldehyde, indicating the full conversion of TMAO. The N‐terminal and the Gcv_T domains generated a neglectable amount of formaldehyde, comparable to the negative control (no enzyme present in solution). Since 7.7 μM of formaldehyde is present in the negative control, it can be concluded that no transformation was performed by these two truncated versions, proving them inactive. Since the single domains are unable to perform the reaction, it is likely that the quaternary structure is crucial for the catalysis. This is further supported by the absence of oligomerization of the purified Gcv_T domain (Figure S6), which elutes as a monomer during the SEC.

### 2.5 pH dependence and TMAO detection threshold

**FIGURE 4:**
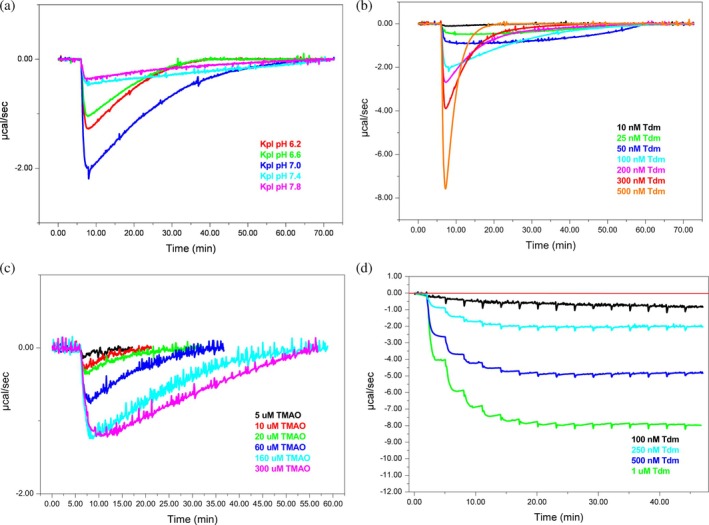
ITC of Tdm. (a) pH dependent activity with KpI at different pH of 6.2 (red), 6.6 (green), 7.0 (blue), 7.4 (light blue), and 7.8 (purple). (b) Enzyme concentration titration from 10 to 500 nM Tdm. (c) Substrate concentration titration from 5 to 300 μM TMAO. (d) Kinetic enzyme assay in multiple‐injection mode with 0.1–1 μM Tdm and 600 μM TMAO.

In single injection mode, the optimal reaction pH and the substrate detection threshold were investigated. Figure 4a shows the peaks associated with the TMAO demethylation carried out in single injection mode with 50 mM KpI at different pH values with TMAO 600 μM and Tdm 100 nM. The exothermic peak of the reaction reaches the highest heat variation value (cal/s) at pH 7.0, indicating that this is the optimal pH for the demethylation reaction. Therefore, the buffer at neutral pH was used for all the reactions.

The minimum enzyme concentration necessary to obtain a calorimetric signal was investigated by ITC. These experiments were performed at a substrate concentration of 600 μM, in single injection mode. As shown in Figure 4b, the protein concentration varied between 10 and 500 nM. Increasing the amount of enzyme leads to an increase in the heat released, indicating that the protein can rapidly metabolize the substrate. The lowest protein concentration that can be exploited for the single‐injection mode is 100 nM, which is usually a really low concentration used in ITC, enhancing the feasibility of this method.

Finally, the detection limit of this assay was explored by keeping the protein concentration fixed at 100 nM in each experiment. The results are displayed in Figure 4c. The detection limit for TMAO was found to be 10 μM. Lower TMAO concentrations did not provide peaks distinguishable from the background noise. The limit of detection is good and relevant for clinical values as it is below the risk threshold in humans and at which the metabolite is generally present in plasma (Dong et al., 2018; Janeiro et al., 2018; Lee et al., 2021; Rexidamu et al., 2019; Wang et al., 2018).

### 2.6 Kinetic characterization of Tdm

Multiple injection mode (Catucci et al., 2019) was used to study the kinetics of the enzymatic reaction. Figure 4d clearly shows that the reaction reaches the maximum velocity after 6–7 injections; therefore, the saturation of the enzyme and the magnitude of the heat change are directly related to the enzyme concentration.

**FIGURE 5:**
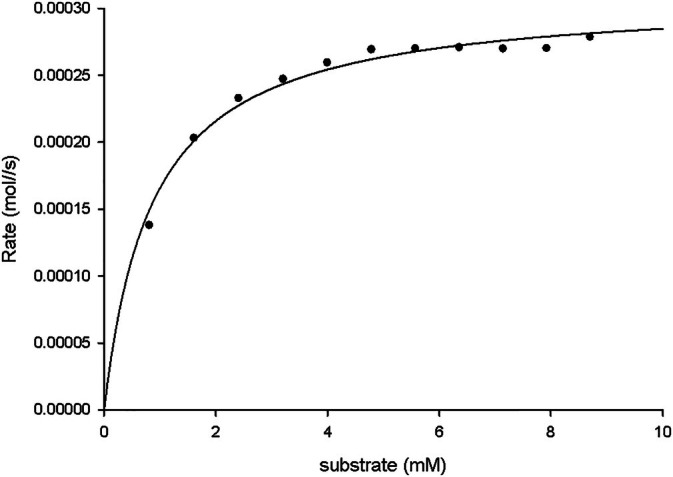
Michaelis–Menten curve fitted with data obtained from the multiple‐injection mode experiment. Michaelis–Menten curve of 100 nM Tdm with multiple injections of 600 μM TMAO.

The data obtained from the multiple injection mode experiments was fitted using Sigma Plot to obtain the Michaelis–Menten parameters, with the goal of identifying the lowest enzyme concentration allowing accurate kinetic measurements and reliable determination of *k_c_*_at_ and *K*_m_ values for use in multi‐injection ITC experiments (Figures 5 and S7), as previously reported (Catucci et al., 2019). As shown in Table 2, 100 nM is the enzyme concentration resulting in the highest *k*_cat_ value. Non‐linear regression of the experimental data produced well‐fitting curves (*R* ^2^ >0.99) (Figures 5 and S7), showing that the method is accurate and reproducible (Table 2).

**TABLE 2:** Michaelis–Menten parameters obtained from the multiple‐injection mode.

Protein concentration	*K*_m_ (mM)	*V*_max_ (μmol/s)	*k*_cat_ (s^−1^)
100 nM	0.866 ± 0.072	0.309 ± 0.004	15.480 ± 0.040

**FIGURE 6:**
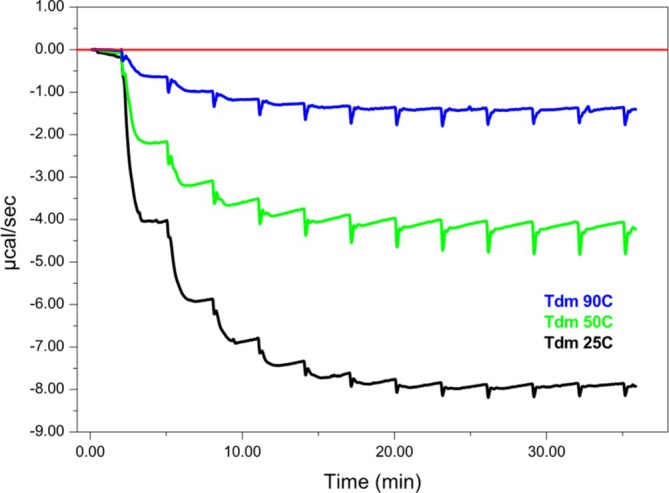
ITC combined with DSC. Multiple‐injection mode titrations for the TMAO demethylation reaction, before after the DSC denaturation, using 100 nM Tdm and 600 μM TMAO.

It was then investigated if the reaction heat observed in multiple‐reaction mode can be directly correlated to the amount of active enzyme in the solution. Therefore, DSC was used to unfold the first domain by performing a temperature increase from 25 to 50°C with a scan rate of 90°C/h and then cooling to 25°C (as it was done in Figure 3c) (Figure 6, green trace). This experiment showed that the unfolding of the first domain only marginally affects the catalytic activity of the enzyme. In a second experiment, the protein was heated to 90°C in DSC with a scan rate of 90°C/h and then cooled down to 25°C. The multiple injection mode ITC experiment performed after this experiment shows that about 21% catalytic activity is left even after complete protein denaturation. This indicates that there is a certain level of stability and resistance to denaturation at the level of the active site. Indeed the residual 21% activity observed after heat denaturation likely reflects partial preservation or local refolding (undetectable by DSC) of structural elements that remain competent for catalysis upon cooling. Since Tdm functions as an oligomer, it is plausible that a fraction of the protein population retains or regains quaternary interactions sufficient to sustain limited enzymatic activity, even after global unfolding. The persistence of these assemblies could stabilize the catalytic core or facilitate partial structural reorganization, allowing a small but measurable fraction of enzymatic function to persist.

## 3 DISCUSSION

In this study, we characterized the stability and the activity of trimethylamine‐*N*‐oxide demethylase using DSC and ITC. Since the crystal structure of Tdm is not known, we used AlphaFold to investigate and understand the three‐dimensional structure of the protein. The software suggests the presence of three distinct domains, against the two originally inferred (Zhu et al., 2014, 2016). The three domains were found to have two known folds: the C‐terminal domain displaying a Gcv_T‐like fold and the DUF1989 at the N‐terminal domains, along with the not known DUF1989‐like third domain. MSA shows high sequence variability in the first DUF1989‐like domain, but its presence in Tdm homologs appears to be ubiquitous. While the model confirms the position of a metal‐binding site for the zinc (Zhu et al., 2016), the originally proposed coordinating residues for the iron seem unlikely. Based on the probable structural proximity of the two metal atoms, we propose that H276, D346, and A344 are the residues coordinating the Fe^2+^. Quaternary structure prediction using AlphaFold suggests that the protein assembles as a trimer of dimers, with part of the oligomerization surface being formed by the DUF1989 domain. This is consistent with Zhu et al.'s findings that single amino acid substitutions in this domain influence oligomerization in solution, thereby confirming the accuracy of our model.

The presence of a Gcv_T domain in Tdm is interesting, especially considering the loss of some critical THF‐binding residues. Additionally, the fact that THF addition to the reaction decreases the amount of formaldehyde released raises questions about the active site position in the structure of Tdm, as well as the reaction mechanism. If the catalysis is linked to the metal atoms, how is the presence of THF in the Gcv_T‐like domain affecting the release of formaldehyde? This point yet requires more analysis. Indeed, the data showing that TMAO demethylase activity is maintained upon denaturation of the more thermally labile unit clearly indicates that only the more thermally stable unit contains the catalytic center, but the full‐length protein in its quaternary structure is strictly required for activity.

We then moved to assess the thermal stability of the protein. To do so a temperature gradient was applied to the enzyme using DSC. Cooling the sample and rescanning did not lead to a different endothermic transition, indicating the irreversibility of the unfolding process of the protein. The data collected by DSC indicate that the denaturation process of the enzyme follows a two‐state mode, with a direct transition from a native to an unfolded polypeptide without intermediate forms for all the protein domains. The thermogram contains two almost completely separated peaks, which is why we hypothesized that there are two independent unfolding events. While the thermogram we obtained from the denaturation of the protein shows two endothermic peaks, the AlphaFold structure prediction indicates the presence of three domains. This fact could be explained by the concomitant denaturation of the two N‐terminal domains, acting as a single cooperative unit (a homodimer), while the other endothermic peak belongs to the denaturation of the Gcv_T domain.

Further calorimetric characterization on the Tdm was done with the use of isothermal calorimetric titration. This fine experimental procedure/analytical method was useful to obtain pH dependence, TMAO detection threshold, and kinetic parameters to develop a methodology to directly measure the demethylation of TMAO, the important CVD biomarker (Janeiro et al., 2018), using only Tdm. For the first two characterization assays the instrument was used in a single injection mode. Initially, we focused on pH dependence, and the results showed clearly that at pH 7 the reaction reached the optimum. The TMAO detection threshold was measured by variation of substrate or protein concentration. First, we identified the enzyme concentration required to generate a measurable calorimetric signal using the ITC. The lowest protein concentration suitable for the single‐injection mode was found to be 100 nM. Then, in the same manner, we addressed the detection limit of TMAO, which was found to be 10 μM. Through the multiple injection mode, we obtained the kinetic parameters of the Tdm that are fully in line with the literature (Catucci et al., 2019). Elevated TMAO levels are linked to an increased risk of cardiovascular disease, stroke, or other pathologies and they are frequently ≥10 μM, while normal plasma TMAO concentrations in healthy individuals are in the range of 2–5 μM. Since Tdm's 10 μM TMAO detection limit roughly matches the lower bound of pathological concentrations, this suggests that the assay has sufficient sensitivity to identify clinically significant elevated TMAO, while it might not be able to differentiate levels below pathological thresholds (Dong et al., 2018; Lee et al., 2021; Rexidamu et al., 2019).

## 4 CONCLUSION

This study aimed to investigate the stability and activity of trimethylamine‐*N*‐oxide demethylase (Tdm) using DSC and ITC, as well as to examine the enzyme's three‐dimensional structure. We identified a previously unreported DUF1989‐like third domain, characterized by high sequence variability and conserved presence across homologs, as revealed through MSAs.

Through calorimetric techniques we characterize the thermal stability, optimal conditions and kinetic parameters of Tdm. Additionally, we successfully developed an ITC assay that allows the determination of both substrate binding and catalytic turnover, offering a reliable tool for future studies involved in the quantification of TMAO, a biomarker with increasing relevance in cardiovascular disease prevention. Beyond its immediate relevance for TMAO detection, the stability and residual activity of Tdm highlight its potential for broader biotechnological applications. The enzyme's robustness and amenability to direct calorimetric monitoring suggest possible uses in biosensor development for clinical diagnostics and as a model for engineering thermostable or oxygen‐independent demethylases.

## 5 MATERIALS AND METHODS

### 5.1 Materials

All solvents and reagents were of analytical grade and were obtained from Sigma Aldrich (St. Louis, Missouri).

### 5.2 Expression and purification of Tdm

The pET28a (+) plasmid vector (Novagen) containing the gene sequence of trimethylamine‐*N*‐oxide demethylase (Tdm) from *M. silvestris* BL2 (GenBank: ACK52488.14), was used to transform a competent strain of *E. coli* BL21 by heat‐shock treatment. The cells were plated on a kanamycin 50 μg/mL LB Agar plate for selection and incubated at 37°C for 16 h. Single colonies were picked from the plate and inoculated in 10 mL of LB medium supplemented with 50 μg/mL of kanamycin. Cultures were grown at 37°C on an orbital shaker (Gallenkamp Environmental Shaker Model 10X 400) at 250 rpm for 15 h. These cultures were used for inoculating 4 flasks (1% v/v preculture) containing 500 mL LB media supplemented with 50 μg/mL of kanamycin. The cultures were incubated on an orbital shaker (Gallenkamp Environmental Shaker Model 10X 400) at 37°C and 250 rpm until the OD reached 0.6. The expression was induced with 0.6 mM of Isopropyl β‐d‐1‐thiogalactopyranoside (IPTG), and the flasks were incubated for 24 h at 25°C and 250 rpm. The cells were harvested through centrifugation in plastic bottles at 4°C (4000 rpm, 20 min, Centrifuge SL 16R, Thermo Fisher). The supernatant was discarded, and the pellet was stored at −20°C for future usage. The enzyme was purified by immobilized metal ion affinity chromatography (IMAC) using a HisTrap HP 5 mL column. The pellet was suspended in Binding Buffer (Tris–HCl pH 7.9, 20 mM imidazole) (4 mL/g) and stirred for 30 min at 4°C. Subsequently, 1 mg/mL of Lysozyme and 0.1 unit/mL of DNAase were added to the cell suspension and stirred for another 25 min. The suspension was then supplemented with 1.5 mM of phenylmethylsulphonyl fluoride and 1 tablet/50 mL of cOmplete protease inhibitor (Roche) and stirred for another 25 min. The cell suspension was sonicated for 5 min (30 s pulse–1 min pause, Misonix Ultrasonic Sonicator, Teltow, Germany) and the lysate was stirred for 1 h at 4°C to let the soluble part separate from the cell debris. The soluble part was recovered by ultracentrifugation at 4°C on a Beckman Coulter Optima L‐90 L using a Ti60 rotor (40,000 rpm, 40 min). The supernatant was loaded on a 5 mL nickel‐ion affinity Cytiva column, equilibrated with Binding Buffer to pH 7.8. The protein was eluted with a gradient elution from 0 to 250 mM of imidazole. The eluted fractions were pooled together for buffer exchange (50 mM Tris–HCl pH 7.8, 200 mM NaCl, 10% glycerol) and concentrated to approximately 35 μM before storage. Fraction purity was assessed by SDS‐PAGE. Protein concentration was assessed using a UV–VIS spectrophotometer at 280 nm (Ɛ_280_ = 73,060) (PerkinElmer Lambda 465, Hellma® absorption cuvette, pathlength 10 mm).

### 5.3 Expression and purification of the N‐terminal and Gcv_T like domains

The truncated versions cloned in the pET 28(+) vector were purchased from Genscript and transformed, expressed, and purified as described above. Fraction purity was assessed by SDS‐PAGE. Protein concentration was assessed using a UV–VIS spectrophotometer at 280 nm, using an extinction coefficient of 29,910 M^−1^ cm^−1^ for the N‐terminal domain and 42,400 M^−1^ cm^−1^ for the Gcv_T‐like domain (Figures S8 and S9).

### 5.4 Analytical size‐exclusion chromatography

The analytical size‐exclusion chromatography was performed on an ÄKTA Start™ chromatography system using a HiLoad™ 16/600 Superdex™ 200 pg gel filtration column (Cytiva). All the experiments have been performed in SEC buffer, containing 50 mM TRIS pH 7.9 and 150 mM NaCl. The calibration of the column was performed using Gel Filtration Calibration Kits (Cytiva) containing proteins with known molecular weights. In detail, 0.5 mL of a solution containing Ferritin, Aldolase, Conalbumin, Ovalbumin, Ribonuclease A, and Aprotinin at the recommended concentrations was injected in the pre‐equilibrated column using a 1 mL loop. Isocratic elution was performed with SEC buffer at 0.5 mL/min. The elution profile was obtained by measuring the absorbance at 280 nm. The SEC of Tdm and the Gcv_T‐like domain has been performed using the same protocol by injecting 0.5 mL of the concentrated fractions eluted from the IMAC.

### 5.5 Metal binding site search with MIB server

MIB (Lin et al., 2016; Lu et al., 2012, 2022) search was done using the AlphaFold (Evans et al., 2022; Jumper et al., 2021) model found on Uniprot (B8EIZ6) as a query. The search was restricted to metal binding sites for Zn^2+^ and Fe^2+^. Metal binding sites were visually inspected to assess their suitability and eventually confirmed by conservation through MSA. Alternative zinc‐binding sites were visually excluded due to the absence of a proper coordination sphere. The only suitable iron‐binding site in proximity to the zinc‐binding site is reported in the results.

### 5.6 BlastP search and MSA

Tdm from *M. silvestris* (WP_012592556.1) was used as a query for a BlastP search against the nr_clustered (experimental) database using a Blosum45 matrix. Sequences with ≤90% query cover were discarded as putatively truncated proteins. Overall, 211 sequences were used for MSA. MSA was performed with the online ClustalW server provided by EMBL_EBI (Madeira et al., 2024). For the MSA display, 7 random sequences were selected in addition to the one of Tdm from *M. silvestris* (8 sequences in total). The image was created using ESPript 3.0 from Multalin (Corpet, 1988).

### 5.7 Differential scanning calorimetry

MicroCal VP‐DSC instrument (Malvern) was used to study the thermal denaturation of Tdm and its isolated domains. The experimental data were analyzed using MicroCal Origin software. All protein samples were analyzed by applying a temperature gradient of 25–90°C with a scan rate of 90°C/h, after 10 min of pre‐scan equilibration. Protein samples (0.6 mg/mL) were maintained in a storage buffer (50 mM Tris–HCl pH 7.8, 200 mM NaCl, 10% glycerol) to provide the best protein stability condition. The same buffer was also used for reference scans.

### 5.8 Formaldehyde production activity assays

Activity assays were performed by incubating 100 mM of TMAO with 1 μM of enzyme in 50 mM KPi buffer, pH 7.0. The reaction was performed for 1 h at 25°C, 500 rpm on a Thermoshaker. The release of formaldehyde was measured using the Fluorimetric Formaldehyde Assay Kit from Cytiva, based on the Hantzsch reaction. Briefly, an aliquot of the reaction mixture was incubated with the buffer reagents for 30 min in the dark. Subsequently, the formaldehyde was quantified by measuring the absorbance of the samples at 368 nm according to the absorption maximum reported by Li et al. (2007) Each sample was measured as a triplicate. An appropriate calibration curve was built using the standard provided with the kit.

### 5.9 Isothermal titration calorimetry

Experiments were carried out in 50 mM KPi pH 7.0 at 25°C using high‐feedback mode with a stirring speed of 350 rpm (Malvern ITC‐200). Trimethylamine‐*N*‐oxide was dissolved in the same buffer. We used a time‐based ITC approach (Catucci et al., 2019). In single injection mode, we used 600 μM TMAO and 100 nM Tdm with different pH (ranging from 6.2 to 7.8). In order to assess the minimum enzyme and substrate detection limit we used 10 nM to 500 nM Tdm with 600 μM TMAO and 5 μM to 300 μM TMAO with 100 nM Tdm, respectively. When operating in multiple injection mode, we tested 0.1–1 μM Tdm and performed different experiments by injecting multiple times the substrate into the cell at a concentration of 600 μM with a spacing of 180 sec so that the instrument was unable to compensate for the heat change avoiding the return to the baseline. A total of 15 injections were performed for each enzyme concentration tested. The goal was to identify the lowest enzyme concentration allowing accurate kinetic measurements and reliable determination of *k*_at_ and *K*_m_ values for use in multi‐injection ITC experiments.

## AUTHOR CONTRIBUTIONS

**Federico Cappa:** Conceptualization; investigation; writing – original draft; methodology. **Nakia Polidori:** Investigation; writing – original draft; methodology; validation. **Daniele Giuriato:** Investigation. **Danilo Correddu:** Investigation. **Arianna Marucco:** Investigation. **Sheila J. Sadeghi:** Supervision; funding acquisition. **Renzo Levi:** Supervision. **Gianluca Catucci:** Conceptualization; investigation; writing – original draft; writing – review and editing; formal analysis. **Gianfranco Gilardi:** Writing – review and editing; writing – original draft; supervision.

## CONFLICT OF INTEREST STATEMENT

The authors declare no conflict of interest.

## Supporting information


**Data S1:** Supporting Information.

## Data Availability

The data that support the findings of this study are available from the corresponding author upon reasonable request.
