# Supplementary material for: Calorimetric characterization of the stability and activity of trimethylamine‐N‐oxide (TMAO) demethylase from Methylocella silvestris BL2
Source: Protein Sci. 2025 Oct 28;34(11):e70364. doi: 10.1002/pro.70364 (PMC12560246; doi:10.1002/pro.70364)
Supplement: Supplementary file 1 — Data S1: Supporting Information. [file PRO-34-e70364-s001.docx]

**Supplementary material**

**Calorimetric characterization of the stability and activity of trimethylamine-N-oxide (TMAO) demethylase from *Methylocella silvestris* BL2**

Federico Cappa^a^, Nakia Polidori^a^, Daniele Giuriato, Danilo Correddu, Arianna Marucco, Sheila J. Sadeghi, Renzo Levi, Gianluca Catucci* and Gianfranco Gilardi*

Department of Life Sciences and Systems Biology, University of Torino, Via Accademia Albertina 13, 10123, Torino, Italy

^a^ Authors share equal contribution

*Corresponding authors:

Gianluca Catucci, e-mail address: [gianluca.catucci@unito.it](mailto:gianluca.catucci@unito.it)

Gianfranco Gilardi, e-mail address: [gianfranco.gilardi@unito.it](mailto:gianfranco.gilardi@unito.it)

**Figure S1. Multiple sequence alignment of *M. silvestris* Tdm with other demethylases.**


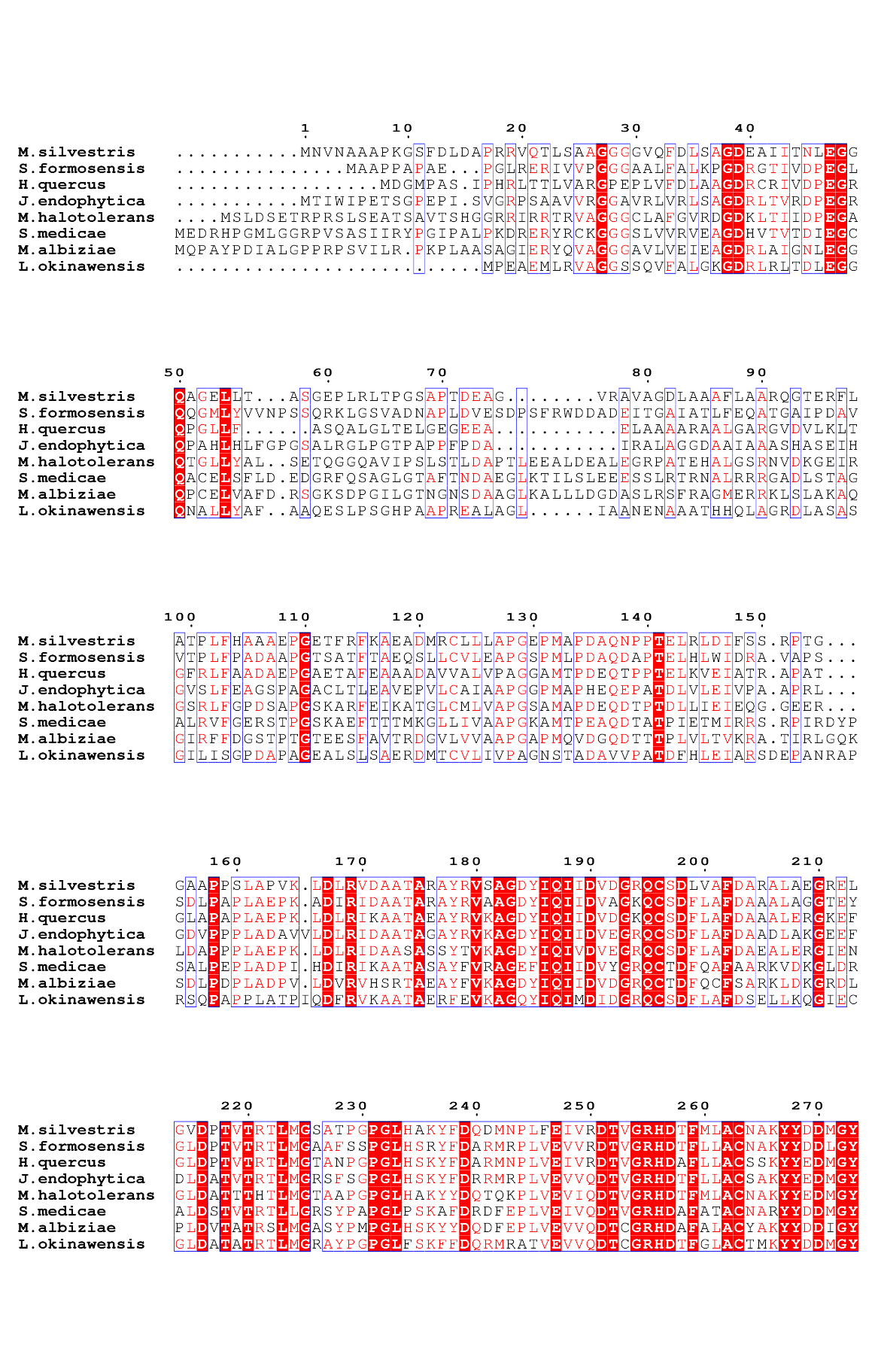


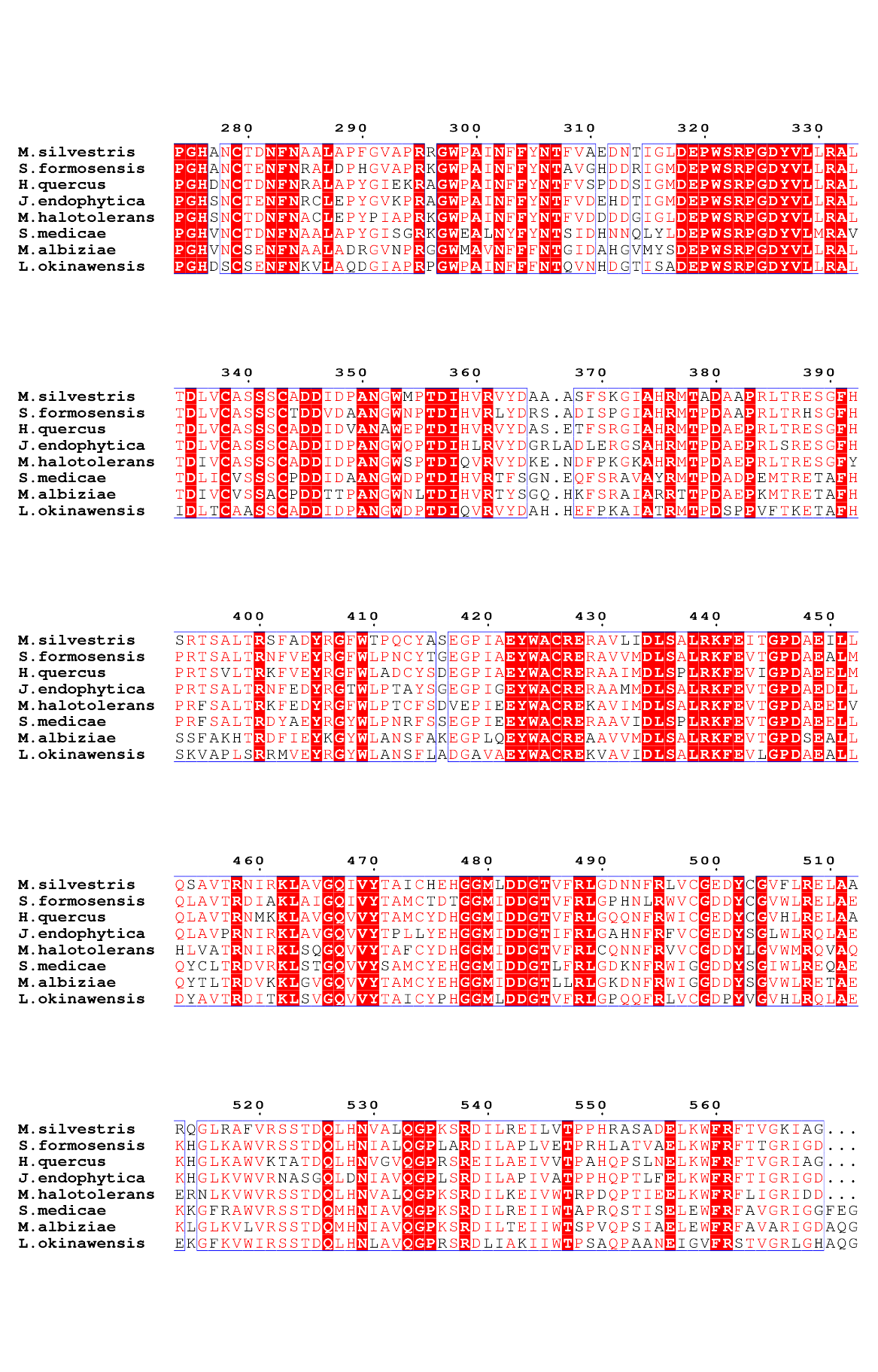


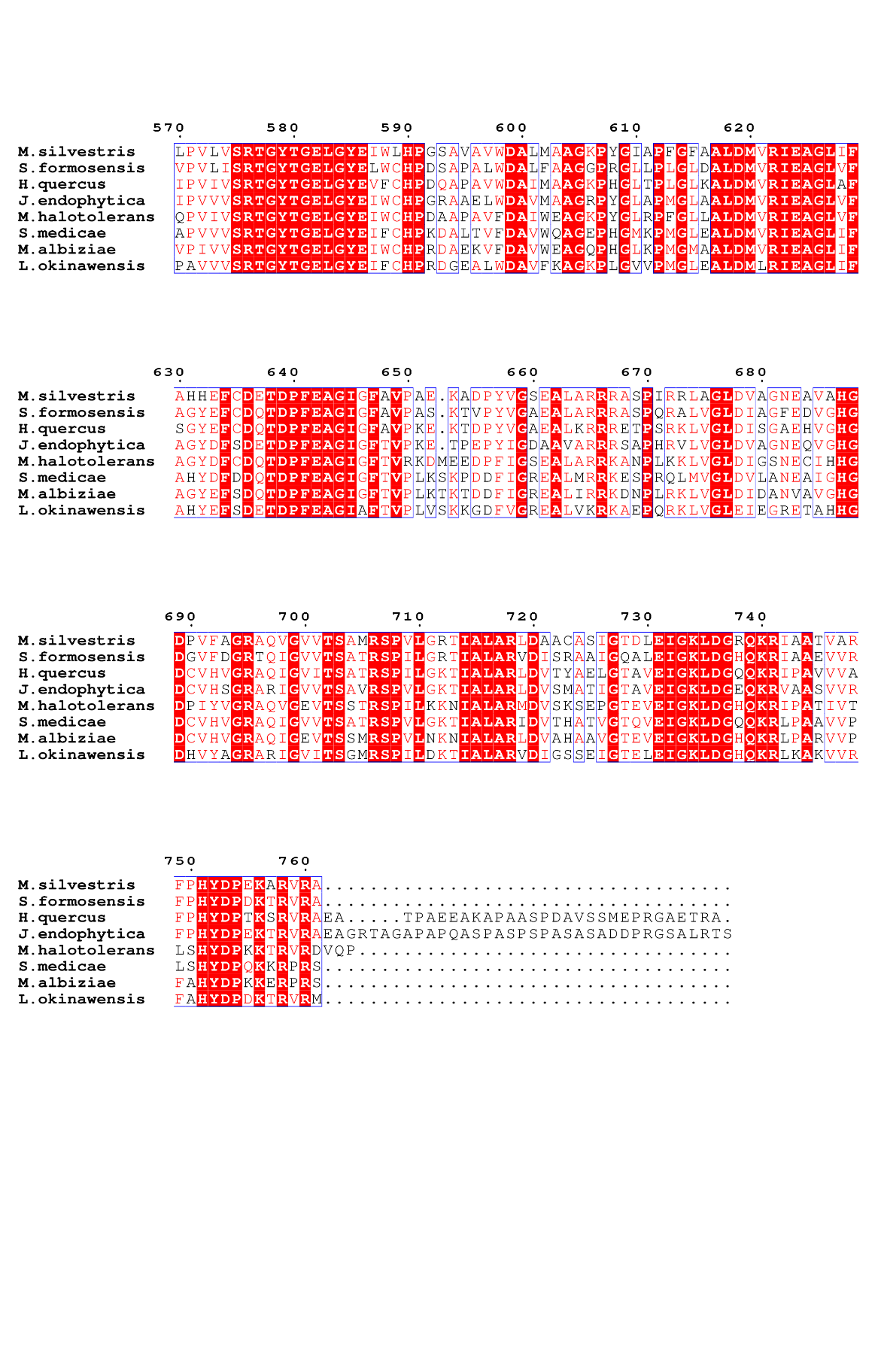


**Figure S2 a) Elution profile of the proteins used for calibration: 1. Ferritin; 2. Aldolase; 3. Conalbumin; 4. Ovalbumin; 5. Ribonuclease A; 6. Aprotinin. b) Calibration curve for the analytical SEC. c) Elution times used for the construction of the calibration curve.**

**
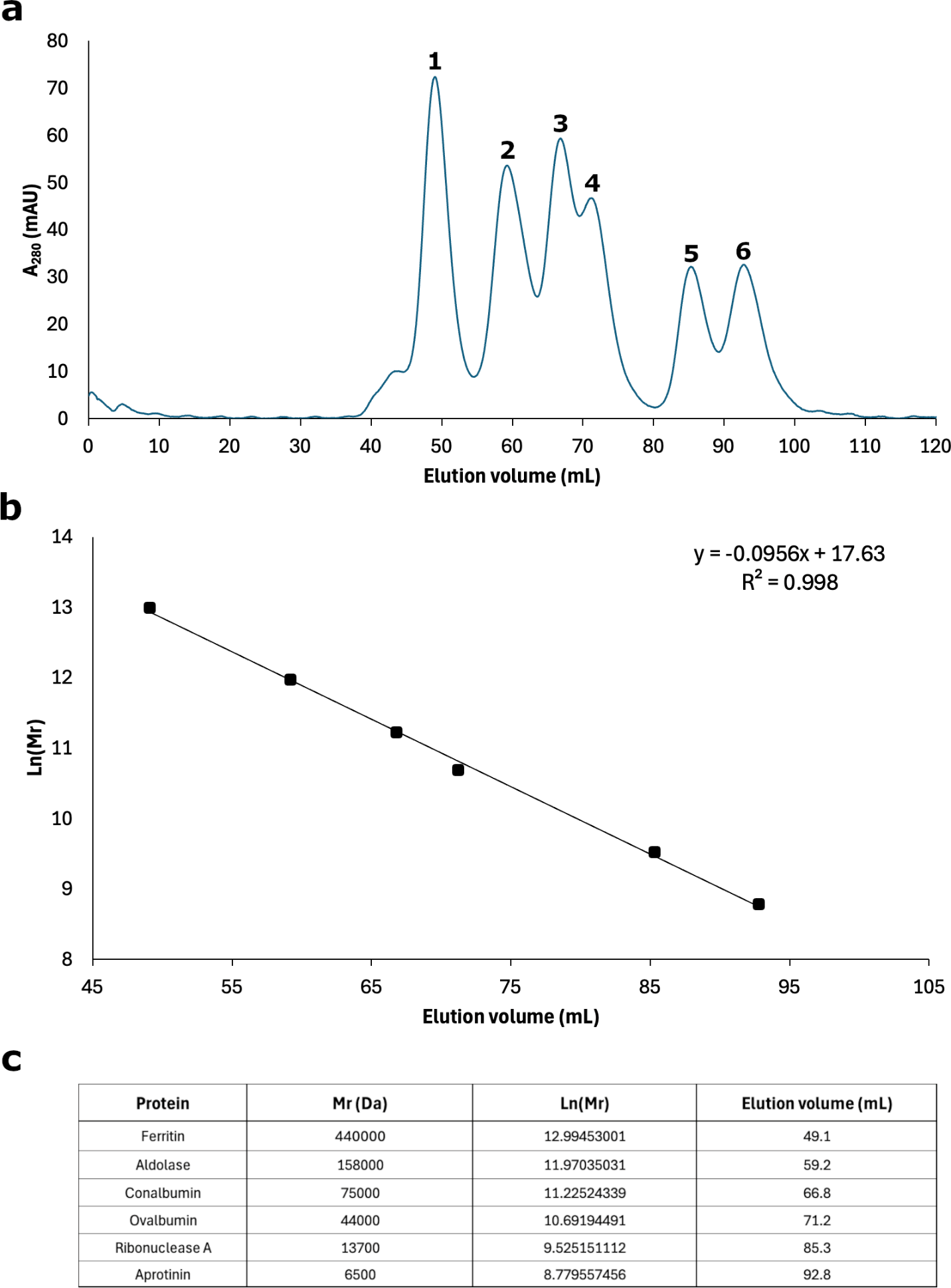
**

**Figure S3 a) Elution profile of Tdm from the Size-Exclusion chromatography on a Hiload 16/600 Superdex 200 pg column. b) SDS-PAGE analysis of the size exclusion chromatography of Tdm.**

**
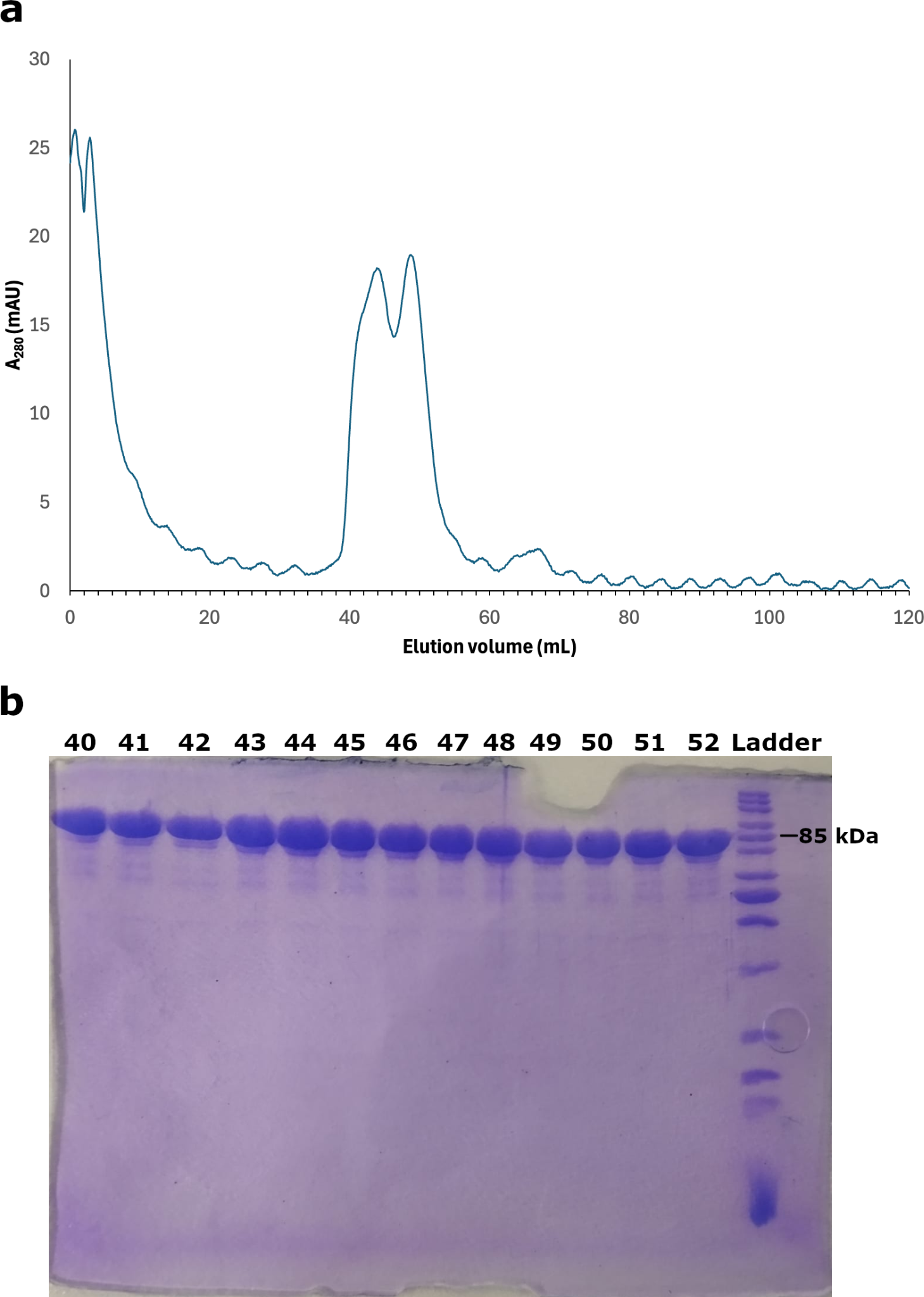
**

**Table S1 Evaluation of the Mr of the Tdm fractions from the analytical SEC.**

| **Elution volume (mL)** | **Ln(Mr)** | **Mr (Da)** | **Mr Monomer (Da)** | **Oligomerization** |
| --- | --- | --- | --- | --- |
| 43.9 | 13.43316 | 682255.84 | 83887.86 | 8.1 |
| 48.8 | 12.96472 | 427077.17 |  | 5.1 |

**Figure S4. SDS-PAGE of purified Tdm.**


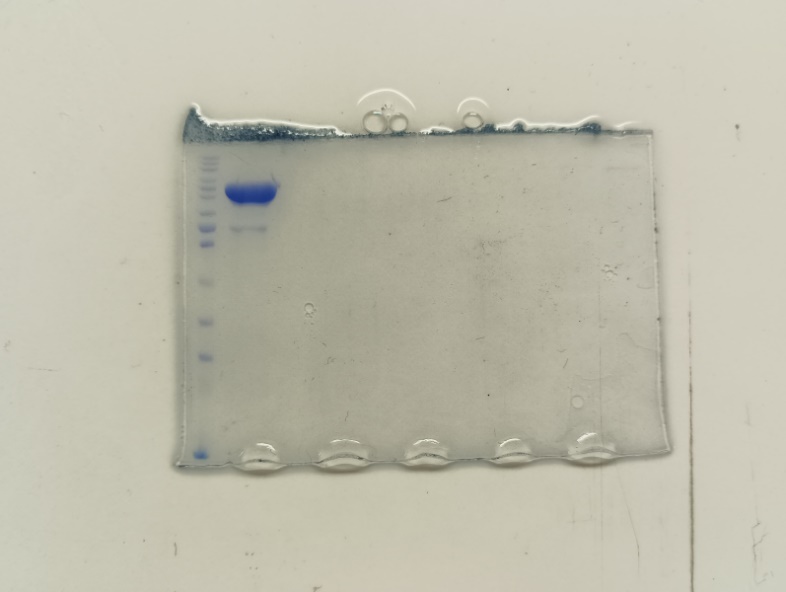


100kDa -

70kDa -

**Figure S5 Calibration curve for the formaldehyde spectrophotometric assay**

**Table S2 Formaldehyde assay of Tdm and its truncated variants**

|  | **A_368_ (AU)** | **Formaldehyde (µM)** |
| --- | --- | --- |
| Tdm | 0.473±0.041 | 107.1 |
| TdmΔ_1-376_ | 0.290±0.029 | 10.8 |
| TdmΔ_377-761_ | 0.284±0.019 | 7.5 |
| Control | 0.283±0.020 | 7.1 |

**Figure S6 a) Elution profile of the GCVT domain from the Size-Exclusion chromatography on a Hiload 16/600 Superdex 200 pg column. b) SDS-PAGE analysis of the size exclusion chromatography of the GCVT domain. c) SDS-PAGE analysis of the size exclusion chromatography of the GCVT domain.**

**
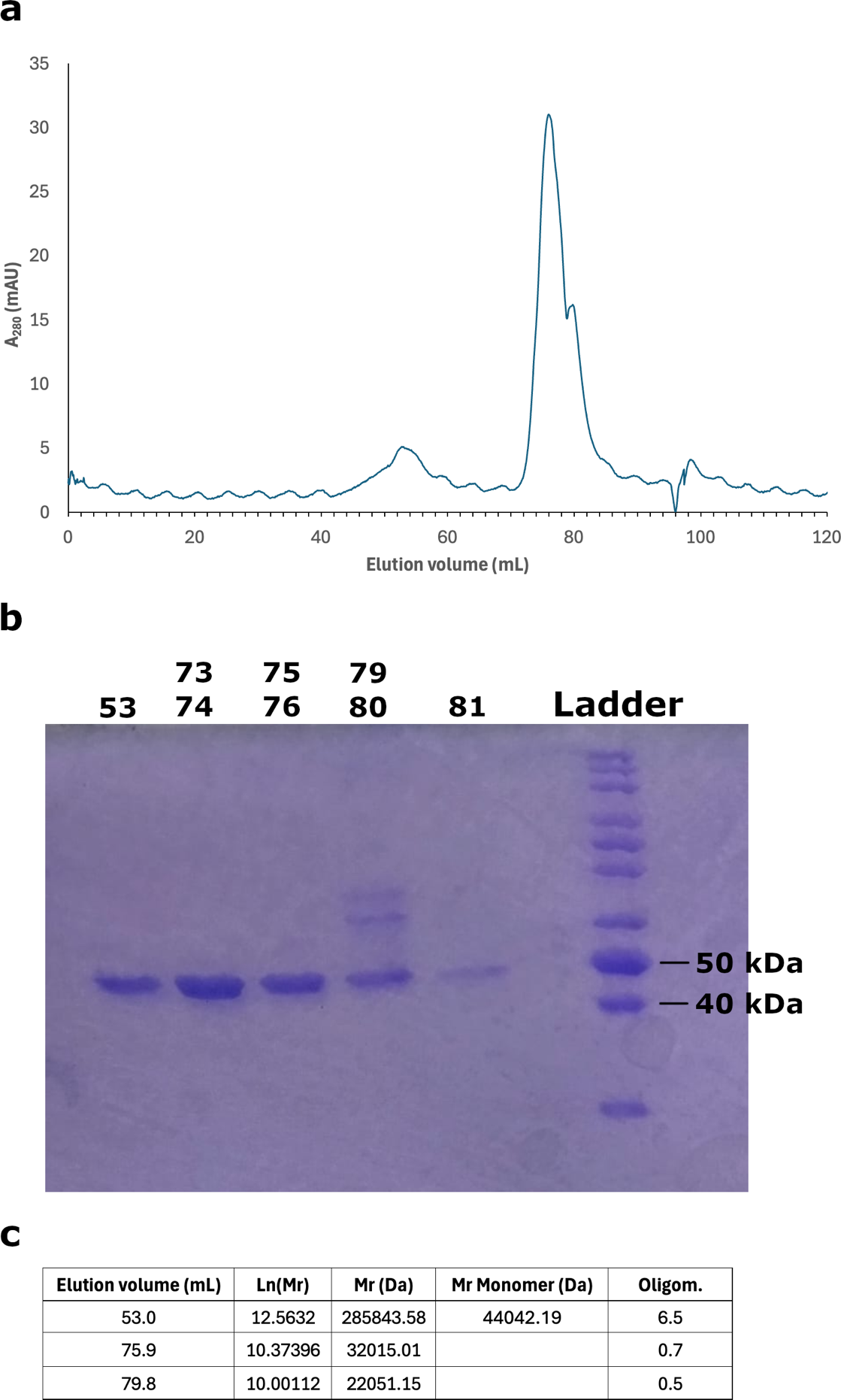
**

**Figure S7 Curves fitted with data obtained from the Multiple-injection mode experiment using multiple injections of 600 µM TMAO and 250 (A), 500 (B), 1000 nM (C) Tdm.**


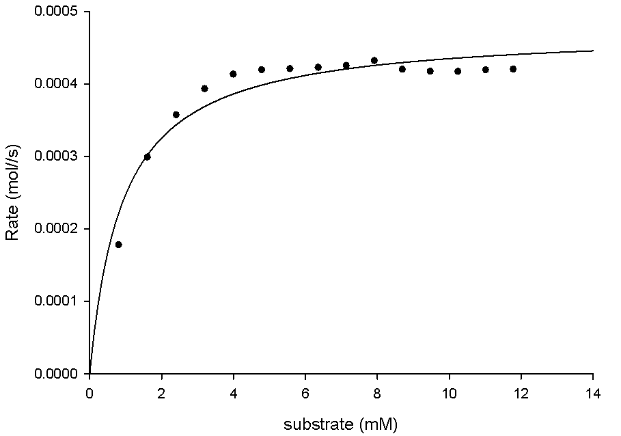

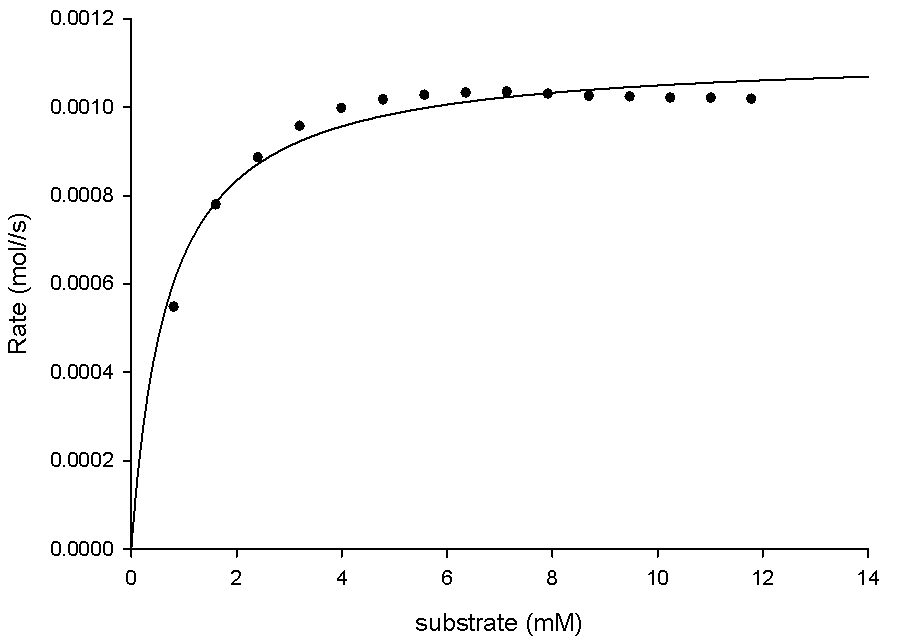

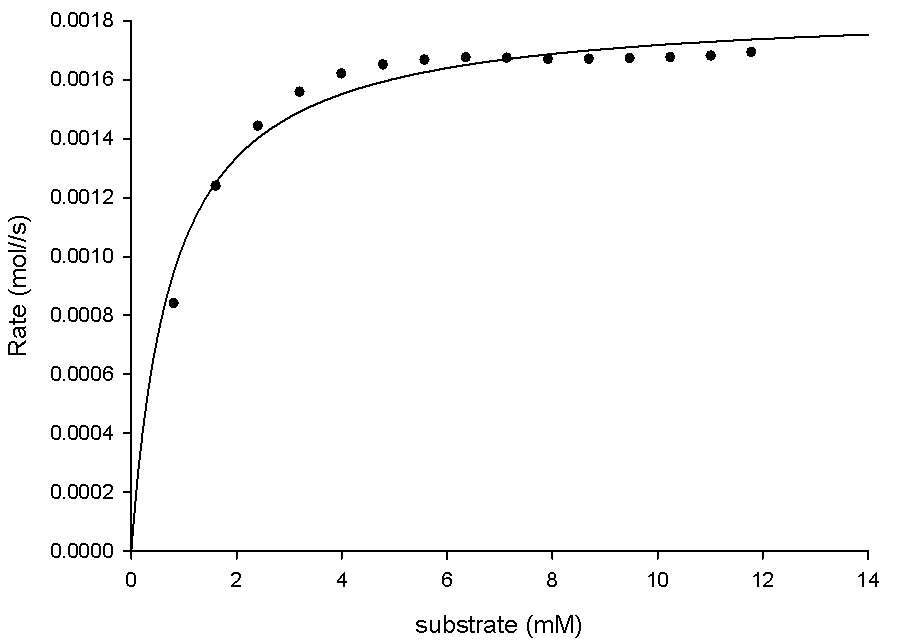


A)

B)

C)

**Figure S8 SDS-PAGE analysis of the IMAC for the GCVT domain purification**

**
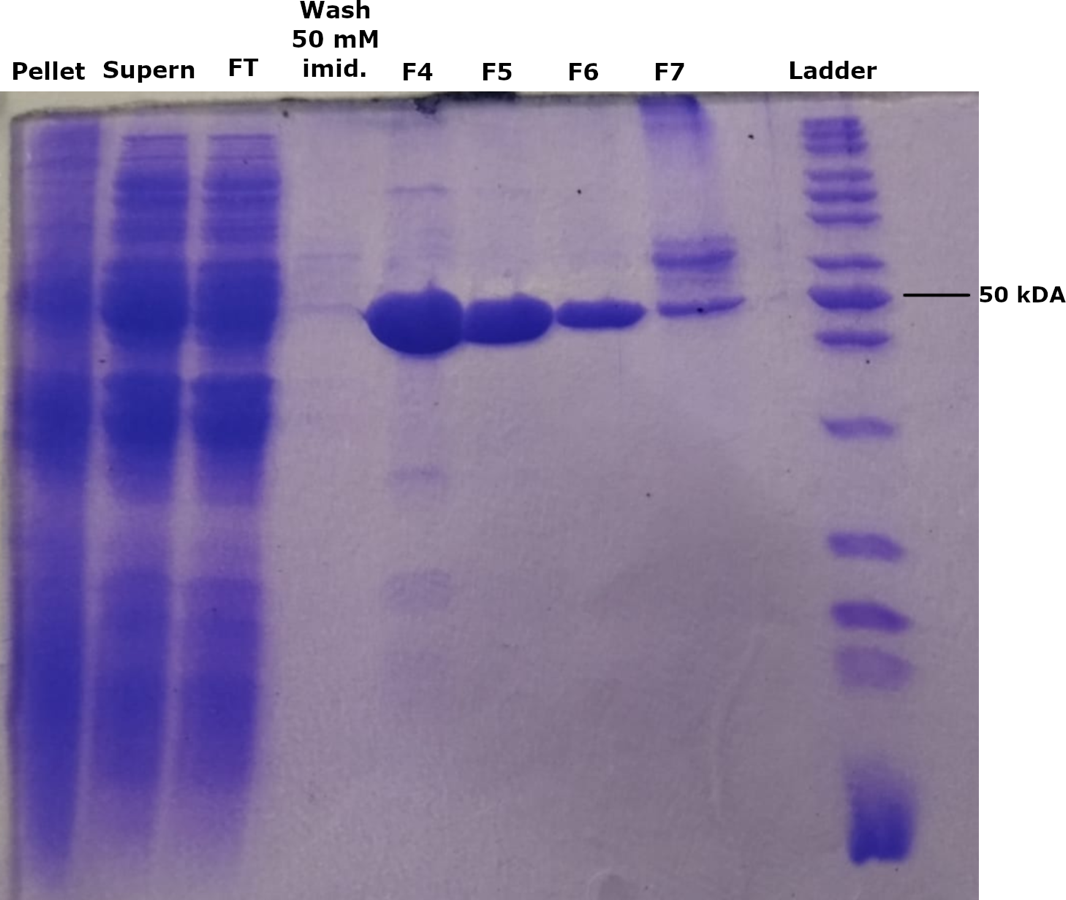
**

**Figure S9 SDS-PAGE analysis of the IMAC for the N-terminal domain purification
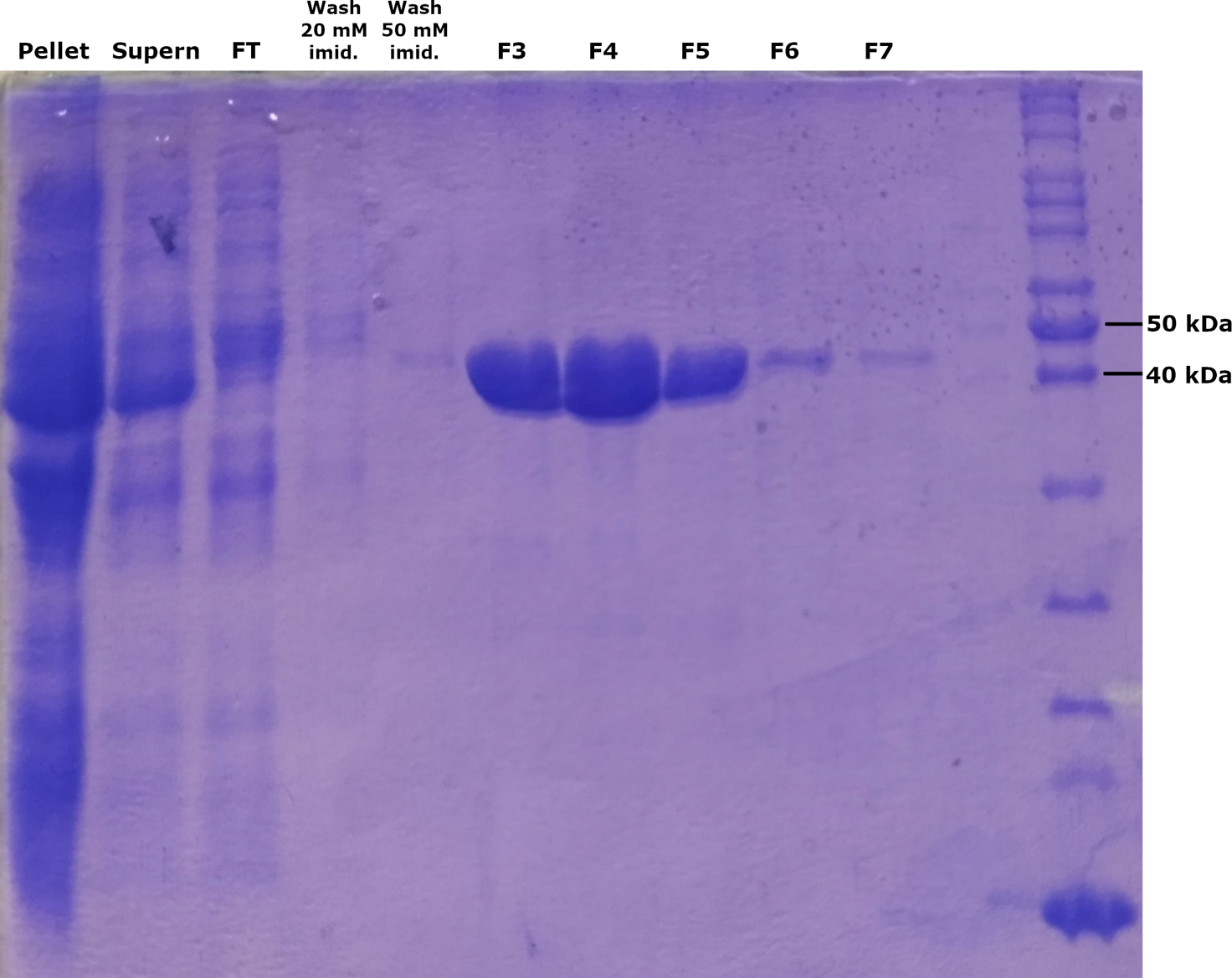
**
